# Genetics of Azoospermia

**DOI:** 10.3390/ijms22063264

**Published:** 2021-03-23

**Authors:** Francesca Cioppi, Viktoria Rosta, Csilla Krausz

**Affiliations:** Department of Biochemical, Experimental and Clinical Sciences “Mario Serio”, University of Florence, 50139 Florence, Italy; francesca.cioppi@unifi.it (F.C.); viktoria.rosta@unifi.it (V.R.)

**Keywords:** azoospermia, infertility, genetics, exome, NGS, NOA, Klinefelter syndrome, Y chromosome microdeletions, CBAVD, congenital hypogonadotropic hypogonadism

## Abstract

Azoospermia affects 1% of men, and it can be due to: (i) hypothalamic-pituitary dysfunction, (ii) primary quantitative spermatogenic disturbances, (iii) urogenital duct obstruction. Known genetic factors contribute to all these categories, and genetic testing is part of the routine diagnostic workup of azoospermic men. The diagnostic yield of genetic tests in azoospermia is different in the different etiological categories, with the highest in Congenital Bilateral Absence of Vas Deferens (90%) and the lowest in Non-Obstructive Azoospermia (NOA) due to primary testicular failure (~30%). Whole-Exome Sequencing allowed the discovery of an increasing number of monogenic defects of NOA with a current list of 38 candidate genes. These genes are of potential clinical relevance for future gene panel-based screening. We classified these genes according to the associated-testicular histology underlying the NOA phenotype. The validation and the discovery of novel NOA genes will radically improve patient management. Interestingly, approximately 37% of candidate genes are shared in human male and female gonadal failure, implying that genetic counselling should be extended also to female family members of NOA patients.

## 1. Introduction

Male infertility is a heterogeneous, multifactorial and complex disorder of the reproductive system, affecting 7–12% of men from the general population [1,2]. Azoospermia (absence of spermatozoa in the ejaculate) can be observed in about 1% of men and the etiology of this condition can be divided into three major categories: (i) hypothalamic–pituitary axis dysfunction, (ii) primary quantitative spermatogenic disturbances, and (iii) urogenital duct obstruction [3]. All of these subgroups can be related to congenital and acquired factors. In case of bilateral distal or proximal obstruction of the ejaculatory ducts, the spermatogenic process is unaffected, and this pathologic condition is termed as Obstructive Azoospermia (OA). On the other hand, primary or secondary testicular failure leads to Non-Obstructive Azoospermia (NOA). NOA is a phenotypic manifestation for which at least three different types of testis histology can be present: (i) Sertoli-Cell-Only Syndrome (SCOS), (ii) Maturation Arrest (MA) at different stages of germ cell maturation (such as Spermatogonial and Spermatocyte Arrest [SGA, SCA]), (iii) hypospermatogenesis.

**Figure 1:**
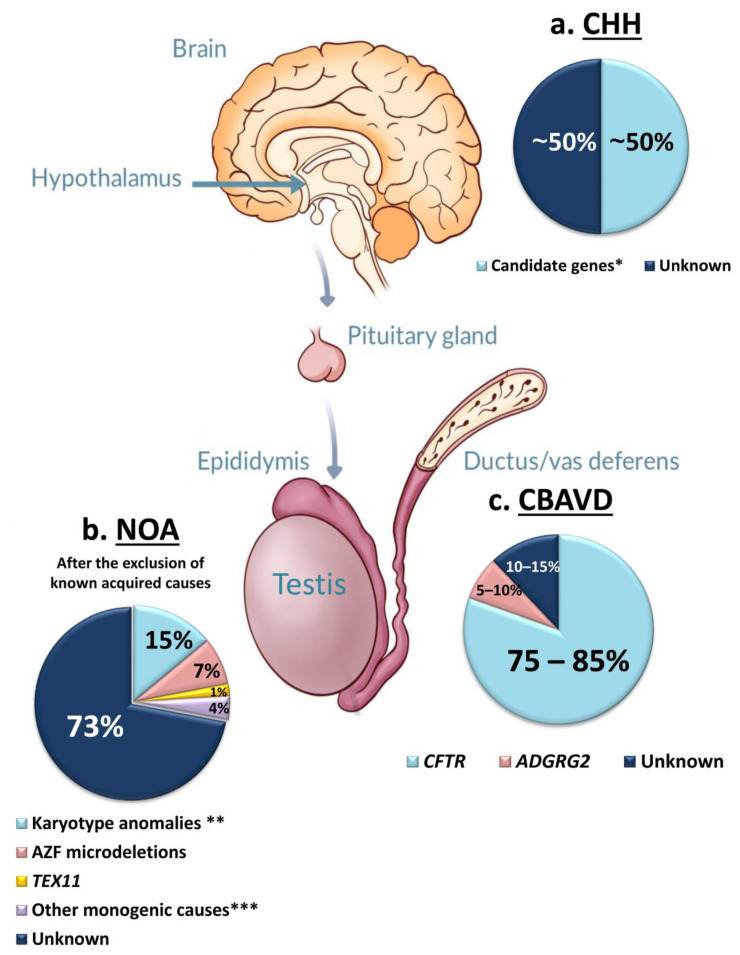
Diagnostic yield of genetic testing in azoospermia with different etiology: (**a**) Congenital Hypogonadotropic Hypogonadism; (**b**) Non-Obstructive Azoospermia due to primary testicular failure, after the exclusion of all know acquired causes; (**c**) Congenital Bilateral Absence of Vas Deferens. Abbreviations: AZF—Azoospermia Factor Region; CBAVD—Congenital Bilateral Absence of Vas Deferens; CHH—Congenital Hypogonadotropic Hypogonadism; NOA—Non-Obstructive Azoospermia; * See Reviews [7,8]; ** 47,XXY Klinefelter syndrome, 46,XX male syndrome, Yq’-‘; *** See articles [9,10,11].

A number of acquired conditions (such as orchitis, cytotoxic treatment, ejaculatory duct obstruction, CNS tumors, systemic diseases etc.) may lead to azoospermia and may account for approximately 35–40% of cases [3]. Concerning the congenital forms, genetic factors play a role in all the above-mentioned etiological categories and some of them are tested as part of the routine diagnostic workup of infertile men [4]. Genetic screening is relevant for its diagnostic value, clinical decision making, and appropriate genetic counselling [5]. In the clinical practice, karyotype abnormalities and Azoospermia Factor (AZF) microdeletions are routinely screened in azoospermic patients [6] due to primary testicular failure. Gene mutation screening based on targeted-gene panels should be recommended when either Congenital Bilateral Absence of Vas Deferens (CBAVD) or Congenital Hypogonadotropic Hypogonadism (CHH) is suspected. The diagnostic yield of the above tests is different in the different etiological categories, the highest is in CBAVD and the lowest in NOA due to primary testicular failure (Figure 1). Next Generation Sequencing (NGS)-based Whole Exome Sequencing (WES) or gene panel sequencing have allowed the identification of a growing number of novel monogenic causes. This review is aimed at providing an overview on genetic factors involved in azoospermia.

## 2. Chromosomal Anomalies Causing Azoospermia

### 2.1. Karyotype Anomalies

#### 2.1.1. Klinefelter Syndrome (47,XXY)

The most common genetic disorder causing NOA is the Klinefelter syndrome (KS), which is characterized by the presence of an extra X chromosome. Although the first description of this pathology was almost 80 years ago [12], due to the extreme heterogeneity in its genetic and clinical presentation, KS continues to pose substantial diagnostic challenges. Its prevalence is 1 out of 600 (0.1–0.2%) in newborn male infants, which rises up to 3–4% among infertile males and 10–12% in azoospermic subjects [13,14]. However, the vast majority of patients (approximately 64%) are still misdiagnosed or remain undiagnosed throughout life [15,16], due to the occurrence of mild forms characterized by paucisymptomatic manifestations [13].

Regarding the karyotype, in the 80–90% of the cases, the pure form with 47,XXY is defined, whereas in the remaining 10–20% of cases higher-grade aneuploidies (48,XXXY or 48,XXYY), structurally abnormal X chromosome (47,iXq,Y) or mosaicisms (47,XXY/46,XY) can be detected [16]. The supernumerary X chromosome could derive from paternal or maternal non-disjunction of the sex chromosomes. This event usually happens either during oogenesis or spermatogenesis, or less frequently (around 3%) during early division of the fertilized oocyte. The failure of chromosome separation at anaphase can occur during meiosis I, meiosis II, or mitosis. Paternal or maternal meiotic aneuploidy appears equally distributed in KS subjects [17], with the difference that paternal non-disjunction can happen only during the first meiotic division, since error in the second meiotic division would result in XX or YY gametes, leading to XXX or XYY zygotes. On the other hand, the age of the mother seems to influence the rate of KS due to post-zygotic origin. In fact, the incidence of KS was 4-fold higher when mothers aged above 40 years were compared to those aged less than 24 years [16]. The reason behind this increase could be that the first three mitotic divisions are controlled by maternal proteins and RNAs, hence, with the advanced maternal age, the chance of mitotic errors increases correspondingly, as well as the possibility of KS of post-zygotic origin [16].

KS has a wide spectrum of clinical manifestations, which includes classical features, such as eunuchoid habitus, hypergonadotropic hypogonadism, gynecomastia, small (volume < 4 mL) firm testes, azoospermia and pervasive neurocognitive deficits.

First of all, mosaic forms are less severe compared to non-mosaic, pure forms of KS. In classic non-mosaic KS patients, the clinical manifestation derives from testis dysfunction (hyalinization), from X–linked gene dosage effect and from the modulatory effect of common polymorphisms [18].

Among typical features are tall stature and eunuchoid habitus. Concerning the former, Short-stature Homeobox-containing gene on chromosome X (SHOX), situated in the pseudoautosomal region 1 (PAR1) on the short arm of the X chromosome (Xp) is involved. Since this gene is implicated in height regulation the presence of three SHOX copies explains the excessive tallness [19].

Another potential genetic modulator is the CAG repeat polymorphism of the Androgen Receptor (AR) gene. CAG repeat length correlates negatively with the function of the Androgen Receptor. It has been investigated in relationship with the broad scale of clinical manifestations seen in KS. The existing literature shows that CAG repeat length has an impact on the androgen-dependent features in KS [13]. Moreover, a positive correlation can be seen with anthropometric parameters such as height, arm span and length, or leg length [20,21,22]. Comorbidities may also manifest as the consequence of gene dosage increase of non-PAR genes, since a certain proportion of them escape X inactivation. It has been suggested that the increased X chromosome gene dosage may alter protein interactome activity with the consequent alteration of cell function [23]. Moreover, there is raising evidence for a role of epigenetic modifications in the clinical manifestations of KS. A globally changed DNA methylation profile, with both hyper- and hypomethylated areas, in the genome of KS patients has been reported [24,25,26]. These epimutations may have regulatory impact on gene transcription with consequent functional effect.

Concerning the fertility potential of KS, it is still not clearly understood how the extra X chromosome affects spermatogenesis. It has been described that extensive fibrosis and hyalinization of the seminiferous tubules occur, with progressive apoptosis of 47,XXY spermatogonia. In the large majority of cases this process results in azoospermia before reaching adulthood [27]. However, spermatogenesis can take place in some seminiferous tubules, which could be explained by two hypotheses: (i) a low level gonosomal mosaicism originated during embryogenesis; (ii) testicular mosaicism due to the loss of the extra X chromosome during mitosis occurring in 47,XXY spermatogonia. Experimental studies based on Fluorescence In Situ Hybridization (FISH), demonstrated that those spermatogonia belonging to tubules with active spermatogenesis were euploid, (46,XY) [28,29]. Hence, these normal diploid germ cells are able to complete the spermatogenic process leading to normal, haploid gametes in the majority of cases [28].

Spermatozoa can be harvested in about 34–44% of KS patients through conventional or micro-Testicular Sperm Extraction (m-TESE) [14,30]. The retrieved spermatozoa can be used for Intra-Cytoplasmic Sperm Injection (ICSI) with an average live birth rate per cycle of 29–43% [14,30]. Since with ageing a progressive loss of germ cells occurs, Rohayem and colleagues [31] investigated the optimal timing of TESE/preventive cryopreservation [32], and predictive factors influencing sperm retrieval rate. The window of opportunity for a higher spermatozoa retrieval success rate was between late adolescence and early adulthood (≥ 15–19 years), with LH ≤ 17.5 U/L and testosterone level ≥ 7.5 nmol/L. No other positive association have been demonstrated with other parameters, such as testicular volumes, serum levels of FSH, Inhibin B, AMH, estradiol. A history of cryptorchidism was found as a negative predictor. On the other hand, Corona and colleagues [30] by performing a meta-analysis did not find an age-related effect on sperm retrieval rate, therefore this issue remains still debated.

With the combination of m-TESE and ICSI, KS men are not considered as infertile anymore, and are able to have their own biological child. Since spermatozoa from KS subjects originates from euploid germ cells, there is no increased risk of having a KS child compared to infertile men with normal karyotype [33]. In fact, more than 200 healthy offspring were born worldwide from KS fathers and only a few cases of KS fetus/newborns were reported [34,35,36].

Given the encouraging data that KS offspring seems not to be affected by the genetic disease of the father, it remains still an open question whether Preimplantation Genetic Diagnosis (PGD) or pre-natal genetic analyses should be recommended [13].

#### 2.1.2. 46,XX Testicular/Ovo-Testicular Disorder of Sex Development

Another karyotype anomaly causing azoospermia is the 46,XX testicular/ovo-testicular Disorder of Sex Development (DSD), also known as 46,XX male syndrome. It was firstly described by De la Chapelle and colleagues in 1964 [37], referring to a rare, heterogeneous clinical condition with an incidence of about 1:20,000–25,000 male newborns [38,39]. The phenotype is largely dependent on the presence or absence of the SRY gene. The SRY gene located on the short arm of the Y chromosome (Yp) is the master gene of male sex determination. The majority of 46,XX testicular–DSD cases are SRY positive (SRY+) (90%) [38,40], thanks to the translocation of SRY-containing segments of the Y chromosome onto the X chromosome during paternal meiosis. These patients usually present with completely differentiated male external and internal genitalia, but decreased testis volume. The minority of 46,XX DSD cases are SRY negative (SRY–). These patients have ambiguous genitalia, poor virilization and ovo-testicular –DSD. The pathogenesis behind this condition could be autosomal gene mutations/over-expression, a gain-of-function in key testicular pathway genes, causing testis differentiation. The most common mechanism is the duplication of SRY-Box 9 (SOX9) gene. Seldomly, duplications of SRY-Box 3 (SOX3) and SRY-Box 10 (SOX10) genes have also been described in 46,XX male cases [41]. Both genes are highly homologous with SOX9, hence their increased expression could mimic SOX9 gene’s function, leading to testis development. Moreover, extremely rare genetic defects, such as large duplication of the Fibroblast growth factor 9 (FGF9) gene [42], or, on the other hand, Loss-of-Function (LoF) mutations in the female pathway genes i.e., in R-spondin 1 (RSPO1) and Wnt Family Member 4 (WNT4) genes have also been associated with 46,XX SRY– DSD cases [43].

A common clinical finding among this group of patients is azoospermia, due to the lack of Y chromosome linked AZF regions, which are essential for physiological spermatogenesis. Hence, in these patients the chance to find spermatozoa in their testicles with sperm harvesting methods is zero. If the couple desires to have children, sperm donation is the only viable option, or adoption. Another frequent remark is the decreased height, corresponding to the absence of growth-regulation genes on the Y chromosome and testosterone level ranging from normal to low with increased FSH and LH levels.

#### 2.1.3. Yq–

The absence of the long arm of the Y chromosome (Yq−) is inevitably linked with azoospermia since it contains crucial genes for spermatogenesis mapping to the AZF regions. These patients present with small testes due to SCOS which recapitulates the complete AZFa deletion phenotype.

### 2.2. Microdeletions of the Y Chromosome: AZF Deletions

**Figure 2:**
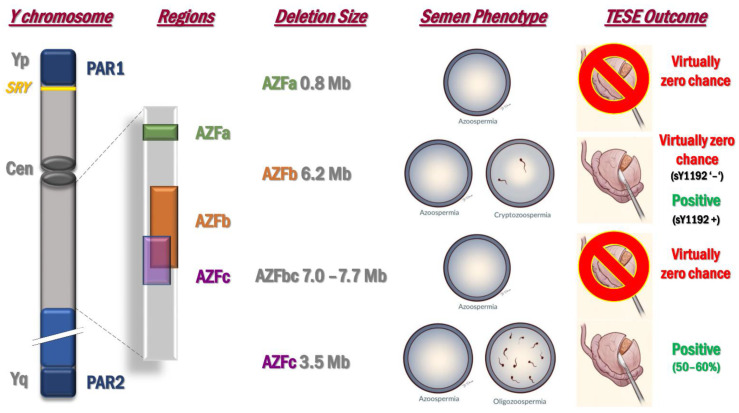
Semen phenotype and TESE outcomes of the different types of AZF microdeletion. Abbreviations: AZF—Azoospermia Factor Region; Cen—centromere; PAR1—Pseudoautosomal Region 1; PAR2—Pseudoautosomal Region 2; *SRY*—Sex-determining Region Y gene; TESE—Testicular Sperm Extraction.

The male sex chromosome is particular for its size, genomic structure, content, and evolutionary trajectory [44,45]. It is haploid, and it precludes recombination with the X chromosome for most of its length (except the two pseudoautosomal regions, PAR1 and PAR2). Moreover, it contains segmental duplications arranged in direct or inverted repeats, and palindromes [44]. The existence of these duplicated sequences allows a mechanism called Non-Allelic Homologous Recombination (NAHR), which might lead to recurrent deletions/duplications affecting the gene dosage on the Y chromosome. The first de novo deletions on the long arm of the Y chromosome (Yq) were described by Tiepolo and Zuffardi in 1976 [46], predicting the presence of Azoospermia Factor(s) (AZF). Further research defined three deletion patterns in proximal, middle and distal Yq11, designated as AZFa, AZFb and AZFc [47]. AZF microdeletions are generally de novo and their origin most likely derives in the testis of the patient’s father. In fact, during meiosis NAHR between sister chromatids may take place leading to AZF deletions [48]. Five Yq fragile sites exist, resulting in the recurrent removal of DNA segments ranging from 0.8 to 7.7 Mb. The most frequently deleted subregion is the AZFc (around 80%), followed by AZFa (around 0.5–4%), AZFb (around 1–5%), and AZFbc, with two different breakpoints (around 1–3%) [49]. The AZF microdeletions are well-known causes of male infertility since AZF regions comprise important spermatogenesis associated genes and gene families [49]. The frequency of complete AZF deletions is 1:4000 in the general population, but it rises up to 5–10% of patients affected by idiopathic NOA [49,50]. The deletion phenotypes for each region are reported in Figure 2. Due to the presence of several genes and their multicopy nature in these regions, it is difficult to understand which genes cause the associated phenotype.

The AZFa region is 792 kb long and contains two ubiquitously expressed, single-copy genes, USP9Y and DDX3Y (former DBY). USP9Y encodes a protein with ubiquitin C-terminal hydrolase activity, which may play an important regulatory role at the level of protein turnover [51]. The isolated absence of this gene is associated with a large spectrum of testis phenotypes, ranging from azoospermia with hypospermatogenesis to normozoospermia [52]. Based on this, USP9Y is more likely to be a fine tuner that improves efficiency, than a gene with an essential function. On the other hand, DDX3Y encodes an ATP-dependent RNA helicase that is a member of the well-conserved DDX3 DEAD Box Helicase family [53]. DDX3Y protein was found predominantly in spermatogonia, whereas its X chromosome homologue, DDX3X, was found to be expressed after meiosis in spermatids. Although isolated mutation or removal of the DDX3Y gene has not been reported, it is likely that the removal of DDX3Y gene is responsible for the AZFa deletion phenotype, which is SCOS. SCOS is characterized by the total absence of germ cells in the testis, low testis volume and high FSH.

Complete AZFb deletion removes a 6.2 Mb DNA segment, including 32 copies of genes and transcription units. These genes are likely to be involved in germ cell maturation since their removal causes spermatogenic arrest. MA is a cessation of germ cell formation mainly at the spermatocyte stage, resulting in azoospermia. MA is typically associated with normal testicular volume and normal gonadotropin levels (LH, FSH), which might mimic obstructive-azoospermia.

The AZFc region involves 12 genes and transcription units, each present in a variable number of copies resulting a total of 32 copies. The clinical manifestation in complete AZFc deletion carriers is largely variable. Spermatozoa may be detected in the ejaculate but typically less than 2 million/mL [50]. Since a progressive decline in sperm count has been observed among these subjects, sperm cryopreservation should be offered to prevent future testis surgery. In those patients who present azoospermia the testis phenotype ranges from SCOS to hypospermatogenesis.

Y chromosome deletion screening is performed in a standardized way, according to the EAA/EMQN guidelines [6]. Two markers are used for each AZF regions besides the control markers of *SRY* and *ZFX/ZFY*. At this regard, it is worth to note that one of the two AZFa markers proposed in the guidelines, contains a polymorphic site in sY84 primer sequence [54]. This SNP (rs72609647) is relatively frequent in the Chinese population while it is yet undetected in the Caucasian population. It is therefore mandatory to use alternative STSs in case of failed amplification of the sY84 marker. For neighboring markers, the “MSY breakpoint mapper” can be consulted (http://breakpointmapper.wi.mit.edu/) [6].

The AZF deletion screening does not only useful for diagnostic purposes but it is also important for TESE prognosis (Figure 2). Carriers of complete AZFa and AZFb deletions have virtually zero chance to find spermatozoa in their testes. In fact, the correct distinction between complete and partial AZFa or AZFb deletions is mandatory through the second step, extension analysis of the AZF deletions. Concerning the AZFb region, Stouffs and colleagues [55] reported two patients with suspected complete AZFb deletion based on the routine first step analysis but with residual sperm production. By performing the extension analysis of the deletion in these patients, the authors were able to amplify a specific distal STS (sY1192) of the AZFb region. This finding indicates that the retention of some copies of multicopy genes such as *PRY*, *RBBMY*, *BPY2*, *DAZ* and some transcription unites may allow residual spermatogenesis and complete maturation. From a clinical point of view, this report called the attention to the importance of testing for sY1192, which is now considered as a decision-making marker: if it is present, TESE might be attempted (Figure 2).

Concerning AZFc microdeletion carriers, spermatozoa can be retrieved through m-TESE from the testicles with a success rate of 50–60% [56]. Data in the literature indicate that a significant proportion of spermatozoa from AZFc microdeletion carriers are nullisomic for sex chromosomes [57,58]. Yq microdeletions could be associated with an overall Y chromosomal instability, which can lead to the formation of 45,X0 cell lines. Karyotype analysis with the search of 45,X0/46,XY mosaicism should be performed on peripheral blood lymphocytes, since mosaicism is considered as a negative predictor of sperm retrieval success [57,59,60,61].

## 3. Monogenic Forms of Azoospermia

All the etiological categories of azoospermia include monogenic causes. Some of them are routinely tested in specific pathological conditions, such as CHH and CBAVD [7,8]. The discovery of monogenic defects of quantitative alterations of spermatogenesis due to primary testicular failure is increasing constantly, but their screening has not been introduced into the diagnostic workup of NOA men, so far. In about 70% of NOA without known acquired causes, the etiology remains unknown, and we refer to it as “idiopathic” NOA (iNOA) (Figure 1). The recent application of NGS (especially in familial cases of NOA) has rapidly increased the number of novel NOA candidate genes. Currently 17 of them have been validated by more than one independent study.

### 3.1. Congenital Hypogonadotropic Hypogonadism (CHH)

CHH is a rare endocrine disease (1:8000 males), caused by the deficient production, secretion or action of the Gonadotropin-Releasing Hormone (GnRH), in the absence of anatomical or functional abnormalities of the hypothalamic-pituitary axis. CHH is a clinically heterogeneous condition covering a wide spectrum of symptoms, where the typical clinical features are delayed puberty and azoospermia. CHH can manifest itself with anosmia/hyposmia (Kallmann Syndrome) or as a normosmic form (nCHH). In addition, non-reproductive features can be recognized in CHH patients, i.e., midline facial defects (cleft lip or palate), unilateral renal agenesis (URA), hearing loss, synkinesia, dental agenesis and short metacarpals [62]. Furthermore, CHH can occur as a part of complex genetic syndromes, such as CHARGE syndrome, Gordon Holmes syndrome and Waardenburg syndrome. CHH is heterogeneous not only clinically but also genetically. To date, more than 40 genes with variable expressivity, penetrance and inheritance have been identified as the genetic cause of the disease and reviewed in two recent articles [7,8]. Currently, through the sequencing of a targeted-gene panel using NGS, a genetic diagnosis is possible in about 50% of cases, and it is expected that novel CHH-associated genes will be discovered by WES analysis in the near future. To note, the main challenge in NGS –based methods is the interpretation of variants of unknown significance (VUSs), hence appropriate tools and expertise are needed for correct interpretation of these findings in the clinical practice.

CHH presents three peculiar features: (1) distinguishing between KS and nCHH is often difficult from a genetic point of view, because mutations in genes involved in GnRH-mediated neuronal migration might result in both forms of CHH [63]. With the exception of certain genes purely associated with Kallmann syndrome or with nCHH, some others (for instance *FGFR1* and *PROKR2*) can be involved in both clinical manifestations, even within the same kindred [7]. (2) CHH is no longer viewed as a Mendelian monogenic disease, since rare variants in two (digenicity) or more (oligogenicity) candidate genes have been found in the same patient, supporting a digenic/oligogenic inheritance in about 20% of cases [63]. Although the oligogenic basis of CHH makes the genotype-phenotype correlation even more complex, it may explain the variable penetrance of the same pathogenic variant within the same family members [7,8]. (3) The traditional view of CHH as a lifelong disease has been changed following the observation of spontaneous remission in patients affected by Kallmann syndrome or nCHH, regardless of type of identified genetic defect [64]. Thus, a periodic suspension of the substitutive testosterone therapy is advised in order to verify the “reversibility”.

In about 80% of CHH patients, spermatogenesis is induced after 9–18 months of gonadotropin treatment [64], and mutations can be transmitted either through spontaneous pregnancy or through Assisted Reproductive Techniques (ART). Overall, the complexity of this disease makes predicting the exact health consequences for the offspring difficult; however, PGD or prenatal diagnosis should be offered to couples, only for syndromic cases and after taking into consideration national legislations.

### 3.2. Congenital Bilateral Absence of Vas Deferens (CBAVD)

CBAVD is a congenital developmental disease (1:1000 males) characterized by the lack of both vas deferens. It may manifest with various clinical features depending on the association or not with other abnormalities of the male urogenital tract, mainly of the seminal vesicles (50% of cases) and the kidneys (renal agenesis occurring in 5–10% of CBAVD patients) [65]. The prevalence of CBAVD in azoospermic men is estimated to be 4–17% and raises up to 25% in case of OA [3,66]. CBAVD associated with agenesis of the seminal vesicles is characterized by a typical semen phenotype, consisting of azoospermia, low semen volume (<1 mL), acid pH (<7). In contrast to CHH, which is a genetically heterogeneous condition (see above), the genetic contribution of CBAVD without kidney anomalies is limited to two genes: (1) Cystic Fibrosis Transmembrane Conductance Regulator (*CFTR*) gene for the 75–80% of cases and (2) Adhesion G Protein-Coupled Receptor G2 (*ADGRG2*) for the 5–10% of cases.

***CFTR*** gene spans 250 kb on the long arm of chromosome 7 (7q31.2), including 27 exons, and encodes for a functional protein of 1480 amino acids. The CFTR protein is involved in the regulation of several ions transporters, including sodium channel, chloride/bicarbonate exchangers, proton exchangers and water channels, and its biallelic dysfunction is responsible for the Cystic Fibrosis (CF) phenotype. Up to now, more than 2000 mutations have been reported in this gene (http://www.genet.sickkids.on.ca/). Disease causing mutations in *CFTR* may affect not only coding regions but also the promoter and deep intronic regions [67,68]. *CFTR* disease-causing alleles can be classified into two main types: (i) “severe” or CF-causing mutations, which are always associated with CF in a homozygous state; (ii) “mild” or non-CF-causing mutations, which have never been observed in CF patients. The presence of two “mild” mutations or one “severe” plus one “mild” allele is causative for CBAVD. Up to 33% of CBAVD subjects of European descendent are compound heterozygous carrying the CF-causing mutation F508del (c.1521_1523delCTT; p.Phe508del) and the non-CF-causing intronic 5T allele (IVS8-5T allele), whose frequency is 4-to-5 times higher in CBAVD patients [69]. However, it is worth noting that some *CFTR* variants are associated with variable expressivity, also called as “variants of varying clinical consequence”, for instance R117H (c.350G > A; p.Arg117His) and IVS8-5T allele. It remains difficult to predict the associated phenotypes in those cases in which one of these variants is in trans with another one of the same type or when it is in trans with a known pathogenic variant [70]. Thus, a genotype involving these variants should be interpreted carefully and reported thoughtfully. For instance, the penetrance of 5T allele is dependent on the status of the adjacent poly TG tract, which usually contains 11, 12, or 13 repeats (c.1210–34TG [11], c.1210–34TG [12], c.1210–34TG [13]). When paired with a known CF-causing variant, 5T and 11TG variants in cis rarely predispose to CBAVD, whereas 5T in cis with 12TG or 13TG confers a risk for CBAVD. Given that 1:10 individuals carry the 5T variant, interpretation of its clinical value should always be performed in the context of the number of associated TG repeats [70].

***ADGRG2*** is an X-*linked* gene encoding an adhesion-class G protein-coupled receptor and is highly expressed in the efferent ducts [71]. In 2016, Patat and colleagues identified 3 hemizygous truncating mutations of the *ADGRG2* gene in 4 out of 26 selected CBAVD subjects with normal kidneys and wild-type *CFTR* gene [72]. Interestingly, *Adgrg2*-mutant mice develop fluid accumulation in the deferent ducts, leading to an obstructive infertility phenotype, which resembles that observed in men with *ADGRG2* mutation [73]. Three other studies have subsequently reported the identification of five new rare variants of *ADGRG2* in 6 CBAVD patients of Asian origin with no pathogenic *CFTR* mutation [74,75,76]. Recently, six novel truncating mutations of *ADGRG2* have been reported in a cohort of 53 French CAVD men carrying none or only one *CFTR* mutation and not presenting with URA [77]. The authors suggested that hemizygous *ADGRG2* mutations are responsible for CBAVD phenotype with normal kidneys, accounting for approximately 20% of unexplained CBAVD cases after a comprehensive analysis of *CFTR* gene [77].

Despite this later discovery, 10–15% of CBAVDs remains without a genetic diagnosis. A portion of these unexplained CBAVD cases coexists with URA, suggesting an early organogenesis disorder. On the contrary, CBAVD related to *CFTR* or *ADGRG2* mutations might be the result of a progressive degeneration that begins later in fetal life and probably continues after birth [65]. The small percentage of CBAVD cases with normal kidneys lacking of genetic diagnosis could be explained by defects in additional genes. For instance, the *SLC9A3* gene has been proposed to be involved in some idiopathic CBAVD cases of Taiwanese origin [78,79]. In addition, epigenetic or environmental factors could contribute to the development of this disease [65].

The clinical management of men affected by CBAVD without renal agenesis must include *CFTR* analysis, which should be based on two steps: (1) a targeted panel of the most commonly known CF-causing mutations in the Caucasian ethnic group, including R117H and 5T allele; (2) a comprehensive screening of *CFTR* for rare point mutations and large rearrangements, when none or only one single mutation has been identified by the first step. If the CBAVD man has no biallelic mutations after comprehensive *CFTR* testing, the analysis of *ADGRG2* is suggested, especially if there is a family history of male infertility. With the high accessibility to NGS-based testing, it could be more cost-effective to perform directly a comprehensive scanning of both genes [65]. Again, the main issue concerns the assignment of a clinical value to VUSs identified by NGS-based methods.

Since the testicular function of CBAVD patients is usually normal, conception of a biological child is possible through TESE combined with ICSI. Given that the carrier frequency of *CFTR* mutations in the Caucasian ethnicity is high (1:25), the *CFTR* panel screening in the female partners is mandatory. If mutations are detected in both partners, the risk of an offspring with CF (classical CF or non-classical CF) is very high and PGD should be advised to the couple.

### 3.3. Primary Testicular Failure

NOA (1:100 males) represent >70% of total azoospermia cases and the majority of them shows primary testicular failure due to an intrinsic defect in the onset or progression of spermatogenesis. As reported in the introduction, NOA can manifest with various testicular histologies, including SCOS, MA at different stages and hypospermatogenesis. SCOS and complete MA are characterized by the absence of haploid cells in the testes, hence the TESE procedure to recover spermatozoa for subsequent ICSI is unsuccessful. In case of incomplete MA, some tubules containing round or later stage spermatids may be found. For this purpose, the development of a pre-TESE diagnostic gene panel would be of high clinical benefit to prevent unnecessary surgery in patients with pure SCOS/MA.

To date, the knowledge of monogenic causes of NOA due to primary testicular failure is limited and none of the current clinical guidelines includes mutational analysis of any NOA genes [9,11]. Given that the spermatogenic process is inherently complex and >2000 genes participate in it [80,81], a high genetic heterogeneity seems to be plausible. So far, 38 candidate genes for iNOA were reported based on the resembling reproductive phenotype in the mouse or on the family segregation (Table 1). Currently, only 17 of them have reached “moderate” or higher grade of clinical evidence, according to the classification criteria proposed by Oud and colleagues [9].

These genes are responsible either for isolated form of NOA or for complex phenotypes where azoospermia may represent one of the clinical symptoms underlying a certain syndrome. In this latter case, we can refer to them as “phenocopy” genes. It is worth noting that, for certain congenital diseases, syndromic features may be non-penetrant in the patient, as well as in the family, or may become evident only with age. For instance, in *FANCA*-mutated patients affected by iNOA, the complex Fanconi Anemia (FA) phenotype may be subtle and need to be specifically assessed to reach a diagnosis of “occult” FA in adulthood (see below; [82]). Thus, in case of pathogenic variants in “phenocopy” genes, a clinical re-evaluation of the patients and relatives should always be part of the diagnostic workup of NOA men.

Very recently, by using STRING analysis of physical and functional protein-protein interactions, Kasak and Laan demonstrated that candidate NOA genes belong to a dense network of “predicted functional partners” [11]. In particular, two distinct clusters of proteic interactions were observed, one for isolated NOA and the other one for syndromic conditions, emphasizing the different etiology underlying the two forms of NOA.

With the exception of *TEX11* and *WNK3* which are X-linked, the remaining candidate genes are mapping to autosomes. Apart from *DMRT1, NR5A1, PLK4* and *WT1* which follow the autosomal dominant inheritance pattern, only recessive mutations lead to quantitative alterations of spermatogenesis (Table 1).

In this paragraph, we will discuss the candidate genes for iNOA (Table 1) according to the different associated-testicular histologies such as SCOS, MA, different types of testis phenotype and undefined testis phenotype.

#### 3.3.1. Candidate Genes for the SCOS Phenotype

**FANCA**: plays an important role in adult-onset syndromic NOA cases. It belongs to the FA pathway [126], taking part in the interstrand crosslink repair and DNA double-strand break (DBS) repair. The *FANCA* gene is the most commonly mutated gene in the genetically heterogeneous FA disorder with variable age of onset [127]. Starting from WES analysis followed by targeted gene sequencing, Krausz and colleagues identified homozygous *FANCA* pathogenic variants in 3/29 selected iNOA patients with SCOS and with slightly altered/borderline hematological parameters [82]. This study underlies the fact that WES may lead to important incidental findings. In this study, undiagnosed FA cases (late-onset FA) before the appearance of other severe clinical manifestations of the disease, including hematological and solid cancers, have been identified. This finding indicates that andrological evaluation, especially in SCOS patients, should not only include hormone measurement but also blood count in order to diagnose “occult” FA cases [82].

***PLK4***: encodes for a protein, which plays a crucial role in the centriole duplication, which is a critical process to be completed before primary spermatocytes can undergo meiosis [128]. It is well-known that homozygous LoF variants in *PLK4* cause microcephaly and chorioretinopathy [129]. However, one man affected by iNOA due to SCOS has been reported as a carrier of a heterozygous 13 bp deletion in the Ser/Thr kinase domain of PLK4 [83]. It is noteworthy that a heterozygous *Plk4*-mutation in mice caused patchy germ cell loss in the testes [130], similar to human, strengthening the potential role of *PLK4* as a dominant cause of SCOS.

***WKN3***: *X-linked* gene with high testis-specific expression, encoding an activator of NKCC1, which has been proposed to cosegregate with the NOA phenotype in a family from Oman [84]. The proband presented with SCOS, but no histological data was available for the affected brothers, thus a phenotypic concordance cannot be established [84]. *Knockout* (KO) *Wnk3* male mice is fertile [125], suggesting that the link between this gene and infertility may not be identical across mouse and men [84]. However, the disruption of the Wnk3-actived *Nkcc1* gene in mice leads to spermatogenic defects resulting in the complete absence of spermatocytes [131].

#### 3.3.2. Candidate Genes for the MA Phenotype

So far, the histological category with the largest number of monogenic defects is represented by the MA phenotype, comprising 22 genes implicated in different stages of spermatogenesis (Table 1b). Six genes involved in meiosis (*MEI1, MEIOB, TEX11, SYCE1, STAG3*, and *SETX*) have been reported to be strictly causative of spermatocyte arrest.

**MEI1**: is implicated in DBS formation, and, along with other NOA causing genes such as *MEIOB, TEX11, TEX15* and *SYCE1*, contributes to the formation and maintenance of the synaptonemal complex and crossovers between homologous chromosomes [11]. Recessive mutations in *MEI1* have been reported in NOA patients exhibiting complete SCA [10,88,89].

**MEIOB**: besides crossover formation and promotion of synapsis during meiosis, it is especially required for DBS repair [132,133]. Men carrying biallelic LoF mutations in this gene also present complete SCA [10,90,91], resulting in an arrest at metaphase I [10]. Interestingly enough, *MEIOB* LoF variants cluster in exon 12, suggesting a hotspot variant region, at least in the Arab/Pakistani population [10,91,134].

***TEX11***: is an X-linked gene belonging to the family of Testis Expressed genes, with the strongest evidence for NOA due to MA [103,104,105]. By using high-resolution array- Comparative Genomic Hybridization (CGH) to screen men with NOA, a recurring deletion of three exons of *TEX11* in two patients has been identified [105]. Furthermore, by sequencing *TEX11* in larger groups of azoospermic men, disease-causing mutations were detected, accounting for more than 1% of NOA cases [104,105,135]. Very recently, Krausz and colleagues demonstrated that defects in the human gene showed a complete metaphase arrest, suggested by a residual spermatocytic development together with the dramatic increase in the number of apoptotic metaphases [10].

**SYCE1**: is a member of the synaptonemal complex, which links homologous chromosomes during prophase I of meiosis. Homozygous mutations in this gene are associated with NOA due to complete SCA [10,100,101]. Meiotic analysis on testicular tissue of *SYCE1* mutation carrier revealed a pachytene arrest (no XY body formation), consistent with the mouse phenotype, with features of unrepaired meiotic DNA DBS in spermatocytes [10].

**STAG3**: is involved not only in DBS repair, but also in the formation of chromosomal axis and cohesion of sister chromatids after DNA replication. Riera-Escamilla and colleagues have demonstrated that biallelic LoF mutations in this gene lead to the persistence of meiotic DBS and to a failure to complete chromosome pairing [92]. Further reports on *STAG3* mutation carriers allowed to classify this gene among those presenting definitive/strong clinical evidence for complete SCA [10,97,98].

**SETX:** is involved in both DNA and RNA processing, and its functional disruption causes syndromic conditions, i.e., Ataxia with Oculomotor Apraxia Type 2 (AOA2) [136] and amyotrophic lateral sclerosis [137]. In the two articles in which testis histology has been described, AOA2 male patients exhibited MA at primary spermatocyte stage [93,94]. This gene represents a typical example of a “phenocopy” gene causing a syndromic disorder in which male and female primary gonadal failure may be one of the clinical signs of the disease. Hence, particular attention must be given to the clinical evaluation of azoospemic patients carrying recessive *SETX* mutations.

In addition to the above reported MA genes, very recently, four novel recessive genes (***ADAD2***, ***TERB1***, ***SHOC1***, and ***MSH4***) have been identified as responsible for MA [10,87,95]. For all these genes the KO mice’s phenotype recapitulates the human phenotype and all of them were validated in independent cohorts of iNOA patients. ADAD2 is a double-stranded RNA binding protein, and it was found to be associated with incomplete SGA in two patients belonging to two independent cohorts [10]. Concerning the other three new genes (*TERB1, SHOC1* and *MSH4*), a complete SCA phenotype was observed in mutated patients. TERB1 is a testis specific telomere-associated protein, which is essential in the regulation of chromosome movement to promote homologous pairing during meiotic prophase I [138]. Meiotic studies of the two *TERB1* variant carriers detected a pachytene arrest [10], which recapitulates the mouse phenotype [138]. Carriers of biallelic defects in *SHOC1* and *MSH4* genes showed a metaphase arrest in the testis that was somewhat less severe compared with the mutant mouse models [10,95].

Besides these recurrently mutated candidate genes, the following 13 genes have been reported only in single studies as causative for MA at different stages with the support of in vitro or in vivo experimental data.

***C14orf39***: encodes a component of the synaptonemal complex and it interacts with SYCE1 via its alfa-helical domain [139]. Very recently, a homozygous frameshift variant of this gene has been identified in two NOA brothers and in their sister affected by Primary Ovarian Insufficiency (POI) [85]. In addition, two different LoF variants have been reported in a homozygous state in two unrelated Chinese NOA-affected patients [85]. All the male *C14orf39* mutation carriers displayed complete SCA [85]. Meiotic analysis on their testicular tissue revealed severe synaptic defects and no XY body formation, indicating that meiosis was arrested at the pachytene-like stage [85]. These observations were strongly supported by the *Six6os1* (the murine orthologue of human *C14orf39*) mutant mouse model, which recapitulated the phenotypes of the NOA and POI individuals [85,139].

**DMC1**: is a meiosis-specific recombinase interacting with several DNA repair proteins in the FA pathway [140], thus it is essential for meiotic homologous recombination and DBS repair [141,142,143]. The lack of this protein results in a block at the leptotene or zygotene stage of meiotic prophase I due to the inability to form synaptonemal complexes [86,142]. Recently, a novel homozygous *DMC1* missense mutation has been identified as the genetic cause of both MA at spermatocyte stage and POI in two siblings from a consanguineous Chinese family [86].

**SPO11:** is essential to initiate meiotic recombination and formation of the synaptonemal complex between homologous chromosomes [144]. To date, a *SPO11* homozygous missense variant has been identified in two brothers with MA [84].

**KASH5** and **RNF212**: are involved in the in the synaptonemal complex and have been recently reported as a potential cause of SCA [84,92]. Importantly, the respective mutant mouse models are supportive to the human genetic data [145,146,147].

**STX2**: is a sulfoglycolipid transporter. In mice, *Stx2* nullizygosity is known to cause spermatogenic failure [148]. In human, a homozygous frameshift mutation has been reported in one Japanese patient presenting with maturation arrest and multinucleated spermatocytes, which have been also observed in mice lacking *Stx2* [99,148].

**XRCC2**: belongs to the FA pathway [126], taking part in the interstrand crosslink repair and DNA DBS repair. Concerning the *XRCC2* gene, a meiosis-specific mutation (p.Leu14Pro) has been proposed as a cause of MA in males and POI in females of a consanguineous Chinese family that not showed other major phenotypesuch as FA [106,149]. The male mouse model with the Xrcc2L14P mutation replicated the human MA phenotype.

**RAD21L1**: is a testis-specific component of the cohesion complex involved in meiotic chromosome pairing and separation [150]. A homozygous stop gain mutation in this gene has been identified in a patient presenting with complete SCA characterized by XY body formation in more than 70% of the tubules indicating completion of synapsis [10]. The authors suggest that *RAD21L1* may be essential for progression beyond meiotic metaphase, but possible not for homologous chromosome synapsis.

**TERB2** and **MAJIN**: TERB2 interacts with TERB1 and MAJIN to form the tripartite meiotic telomere complex (MTC), which has been shown in mouse models to be necessary for the completion of meiosis and both male and female fertility [151,152]. Compound heterozygous frameshift variants in *TERB2* gene have been found to cosegregate with MA phenotype in a non-consanguineous family in which 3 sons were affected [87]. Concerning the *MAJIN* gene, a rare homozygous missense variant has been identified in one sporadic case affected by germ cell maturation arrest with occasional post-meiotic round spermatids in 2–4% of tubules [87].

***SPINK2***: encodes a member of the family of serine protease inhibitors of the Kazal type, which is necessary to neutralize the action of acrosomal proteases shortly after their synthesis and before they can be safely stored in the acrosome [96,153]. A homozygous truncating mutation in the *SPINK2* gene has been reported cosegregating with NOA phenotype due to the arrest of spermatid differentiation at the round stage [96]. Homozygous KO animals also suffered from azoospermia, thus confirming the potential implication of *SPINK2* in NOA [96].

**TDRD7**: is a component of chromatoid bodies contributing to the post-transcriptional regulation of specific mRNAs and it plays a role in the development of haploid spermatids in adulthood [154]. Recently, biallelic LoF variants in this gene were reported in two consanguineous Chinese families to cause a rare syndrome combining congenital cataract and NOA due to MA [102].

**ZMYND15**: is a testis-specific transcriptional repressor that controls normal temporal expression of haploid genes during spermiogenesis [155]. Ahyan and colleagues have proposed this gene as a cause of recessive azoospermia in two consanguineous Turkish families [107]. Its functional disruption results in maturation arrest at the spermatid stage [107], suggesting that NOA can also be induced by post-meiotic defects.

#### 3.3.3. Candidate Genes Associated with Different Types of Testicular Phenotype

Pathogenic mutations in some candidate NOA genes are not associated with a clear-cut testicular phenotype and different testis histology, ranging from SCOS to hypospermatogenesis, can be observed in different mutation carriers (Table 1c).

***DMRT1***: encodes a transcription factor that plays a key role in testis differentiation. Its monoallelic disruption is well-known to be associated with syndromic and non-syndromic forms of XY gonadal dysgenesis [156,157]. By performing genome-wide array-CGH, four deletions spanning the *DMRT1* gene were reported in a total of five men from independent cohorts affected by isolated NOA [108]. Both SCOS and SCA testicular phenotypes have been found to cosegregate with the deletion of this gene [108]. Very recently, a heterozygous deletion of exons 1 and 2, resulting in the removal of the entire DM/DNA-binding domain, has been observed in a case of incomplete SGA [10]. These findings suggest that besides XY gonadal dysgenesis, *DMRT1* deletions may play a role in the occurrence of NOA.

***FANCM***: this testis-enhanced gene belongs to the FA pathway. This is one of the few genes in the pathway that does not cause the FA phenotype [158,159]. It plays a crucial role in major cellular functions, including DNA replication/repair and anti-crossover to maintain genomic stability [160]. Kasak and colleagues reported that biallelic LoF variants in this gene are the most likely cause of the SCOS phenotype diagnosed in four patients [110]. Recently, a homozygous frameshift mutation in *FANCM* was found cosegregating with male infertility in a consanguineous Pakistani family, in which three brothers presented with either oligoasthenozoospermia or azoospermia [109]. Hence, the spectrum of the seminal phenotype in patients with biallelic truncating *FANCM* variants seems to be widespread, implying that some mutations may lead to milder phenotypes. Interestingly, the *Fancm*-mutant mice displayed SCO tubules and a progressive loss of germ cells [109,161,162], which may derive from the defective repair of interstrand crosslink occurring during DNA replication of the germ cells. These features strengthen the link between *FANCM* mutations and SCOS phenotype in humans.

***NANOS2***: encodes an RNA-binding protein that contribute to the maintenance of the spermatogonial stem cell population and suppression of meiotic entry [163]. *Nanos2* KO models lead to male-specific complete germ cell loss in both Drosophila and mouse [164]. After the first study on the role of *NANOS2* as a potential cause of SCOS phenotype [165], a homozygous mutation in this gene was recently reported to cosegregate with SCOS [84] in two brothers from a consanguineous family. However, one additional sporadic patient carrying a homozygous start loss variant presented with MA [84], questioning the clear-cut relation with the SCOS phenotype.

***TEX14*** and ***TEX15*** belong to the family of Testis Expressed genes. TEX14 is required for the formation/maintaining of intercellular bridges (IC) in vertebrate germ cells, which are essential for meiosis during spermatogenesis [166]. This gene appears to be exclusively expressed in the human and mouse testis and it is conserved among mammals [90]. Severe spermatocyte depletion was observed in *Tex14* KO mice [166]. In humans, recessive mutations in *TEX14* were found to be strong candidates for NOA with testis histology ranging from SCOS to early MA phenotype with negative TESE outcome [10,84,90]. TEX15 plays a key role in the recruitment of DNA repair proteins into DBS locations. It is worth noting that, unlike *TEX14* which is a negative predictor of sperm retrieval in testis, *TEX15* has been found mutated both in patients with NOA and crypto/oligozoospermia [103,118,119,167].

***NR5A1*** and ***WT1***: encode the Steroidogenic factor 1 (SF1) and the Wilms’ tumor protein, respectively, which are well-known and functionally interacting transcription factors implicated in gonadal development in both sexes. In fact, WT1 modulates SF1 in a sex-specific manner [168]. Mutations in *NR5A1* and *WT1* primarily cause AD syndromic phenotypes (reviewed in [169]) associated with NOA due to SCOS or MA [112,115]. Mutations in *NR5A1* are well-known to cause AD primary adrenal insufficiency and 46, XY disorders of sexual development (DSD) [170], besides hypospadias, bilateral anorchia and micropenis in addition to women with POI [171]. Some pathogenic *NR5A1* variants are responsible only for NOA phenotype, without any clearly identifiable developmental defects in the testis [112,113,115]. Mutated or deleted *WT1* leads to a spectrum of congenital defects in kidneys and genitalia (such asDenys-Drash syndrome, Wilms’ tumor, nephropathy), including DSD disorder [172]. To note, pathogenic *WT1* missense variants have also been reported in patients with the solely diagnosis of NOA without malformations in the genitourinary tract [120,121,122]. We can conclude that defects in both genes are characterized by variable expressivity and incomplete penetrance, including asymptomatic family members [173,174], thus the clinical management of these patients needs careful evaluation. In particular, defects in these genes could be suspected in patients presenting hypospadias, cryptorchidism, and other signs of a congenital testicular damage [170,175].

**TAF4B**: is a ubiquitous transcription factor acting as a gene-selective coactivator, whose homozygous truncating variants lead to the NOA/oligozoospermia phenotype in a Turkish consanguineous family [107]. Interestingly, *Taf4b* KO mice are subfertile with extensive pre-meiotic germ cell loss due to altered differentiation and self-renewal of the spermatogonial stem cell pool [107].

**TDRD9**: is essential for silencing of Line-1 (L1) retrotransposon in the male germ line, both in mouse and in human, for enabling fertility [117,176]. A homozygous frameshift mutation in the *TDRD9* gene has been identified in five men of a large consanguineous Bedouin family, diagnosed as having cryptozoospermia/azoospermia due to incomplete MA [117]. The authors conclude that the mutation is the cause of the spermatogenic impairment, resembling that observed in the *Tdrd9* knockout mice, without any involvement in female infertility [117,176].

***M1AP***: encodes a protein that is likely to function in meiotic progression. Recently, *M1AP* has been found to be mutated in patients affected by NOA due to MA [111]. Data from four independent cohorts revealed that biallelic LoF mutations of *M1AP* are associated with a variable spectrum of severely impaired spermatogenesis, mostly meiotic arrest resulting in azoospermia, but occasionally spermatids and rarely a few spermatozoa in the semen were observed. A similar phenotype has been described for mice with disruption of *M1ap* [111].

#### 3.3.4. Candidate Genes for iNOA with Undefined Testicular Phenotype

Novel promising candidate genes for NOA have been recently reported, without providing the clinical histological data of the carriers, precluding their classification into the above-mentioned testis phenotype categories (Table 1d).

**MCM8**: has been recently suggested to interact with members of FA pathway in crosslink repair during replication [177]. This gene proved to be crucial for gonadal development and maintenance in humans, both males and females. In fact, homozygous *MCM8* mutations resulting in genetic instability due to meiotic DNA repair defect have been demonstrated to be the cause of NOA in males and POI in females [123].

***PSMC3IP***: encodes a critical coactivator of DMC1 and RAD51 proteins [178,179,180] and it is implicated in meiotic recombination. In a consanguineous Yemeni family, a homozygous *PSMC3IP* stop gain mutation deleting the C-terminal portion of the protein has been found to cosegregate with POI and NOA phenotypes [124]. It was found that PSMC3IP protein deprived of C-terminal domain fails to associate with the DMC1 and RAD51 proteins required for homologous recombination [179]. In mice, the absence of Psmc3ip protein leads to the arrest at the primary spermatocyte stage, indicating a block at meiosis I [181].

## 4. Common Monogenic Defects in Male and Female Primary Gonadal Failure

An emerging issue in the field of human reproduction concerns common genetic factors between male and female infertility. Several genes causing NOA in males are also considered to be involved in female reproduction, leading to the POI phenotype (Table 1). POI is a heterogeneous disorder characterized by primary or secondary amenorrhea in women younger than 40 years of age [182]. The three main mechanisms leading to POI can be: (i) an impaired formation of primordial follicles leading to a reduced number of their pool; (ii) an impaired recruitment and/or an altered maturation of the follicles; (iii) an increased follicular atresia [183]. Isolated or non-syndromic POI is recognized in ~1–2% of women and it has a heterogeneous genetic basis [182,184], which accounts for approximately 20–25% of POI patients [185]. Given both the similar incidence and the identification of shared genetic factors, POI can be considered as the corresponding female phenotype of oligo/azoospermia. In support of this, similarly to NOA, POI is associated with a significantly higher morbidity in respect to females with physiological menopause [182]. In addition, an increased risk of osteoporosis, cardiovascular diseases, type 2 diabetes has also been reported [182,186], making this condition a public health problem [186]. Candidate genes involved in both POI and NOA pedigrees are mainly related to DNA damage repair (*FANCM*, *FANCA*, *XRCC2*, *MCM8*), homologous recombination and meiosis (*STAG3*, *SYCE1*, *C14orf39*, *MSH4*, *PSMC3IP*, *DMC1*, and *MEIOB*), along with the transcriptional activator involved in sex determination, such as *NR5A1*. In addition, candidate genes of syndromic male and female infertility are *SETX* and *WT1*. It is noteworthy that the inheritance pattern of POI is complex, since at least two mutations in distinct candidate genes have been recognized in 42% of patients, arguing in favor of a oligogenic nature [187].

***FANCM***: has been first reported in two Finnish sisters affected by non-syndromic POI [188]. The homozygous stop gain *FANCM* mutation identified in this family may provoke meiotic defects leading to a depleted follicular stock, as in *Fancm −/−* mice [188]. Interestingly, the parents and the 20-years-old brother carrying the mutation in a heterozygous state were healthy, confirming the recessive inheritance mode of *FANCM*. Notably, the same homozygous nonsense variant in *FANCM* was identified in an Estonian NOA-affected case [110].

***FANCA***: two rare heterozygous missense variants have been recently identified by WES in two unrelated females, one with primary amenorrhea and the other one with non-syndromic POI [189]. In order to verify the potential pathogenic effect of heterozygous mutations, the authors performed in vitro studies showing that the mutations in a heterozygous state partial affect FANCA expression levels and its signaling pathways [189]. Heterozygous mutated female mice (*Fanca+/−*) showed reduced fertility and progressive decline of follicles with aging when compared with the wild-type female mice, suggesting a possible contribution of *FANCA* haploinsufficiency to POI [189]. However, given that the mode of transmission is autosomal recessive for most of the meiosis or DNA repair genes, especially for genes of the Fanconi Anemia pathway, it is still debated whether a causal link between heterozygous *FANCA* variants and POI may exist [190].

***XRCC2***: as mentioned in the previous paragraph, a homozygous missense variant (p.Leu14Pro) of the gene has been proposed as a meiosis-specific mutation causing both NOA and POI [106,149]. Homozygous female mice for the *Xrcc2-L14P* allele exhibited reproductive disorders that were consistent with POI [106].

***MCM8***: similarly to the above gene, mutations in *MCM8* have been reported as a recessive cause of both isolated and syndromic POI [123,191,192,193,194,195,196] and isolated NOA [123]. *Mcm8*-deficient mice have small gonads and are infertile (female and male) [197], as observed both in women and in men carrying homozygous *MCM8* inactivating variants [123,191,192,193,194,195,196]. KO mice models suggested that in both sexes the gonadal function impairs with aging [197].

***STAG3*** was first described as a POI gene in 2014 [198], and since than recessive high-impact variants have been described as a rare but recurrent cause of non-syndromic POI [198,199,200,201,202,203,204]. Very recently, a homozygous *STAG3* missense variant cosegregated with the infertility phenotype in a consanguineous family including a proband with POI and her brother with NOA [97]. These findings are consistent with *Stag3* KO mice, showing an early prophase I arrest and apoptosis in both male and female germ cells [205].

***SYCE1***: the first report identified a homozygous point mutation in a 13-member-family in which two sisters born to consanguineous parents suffered from POI [206]. Hernandez Lopez and colleagues [207] have demonstrated that the homozygous state of the previously described point mutation severely affects homologous chromosome synapsis, which would most probably account for the observed gametogenesis failure both in male and in female mice. As stated in the previous paragraph, a similar observation was made in male carriers [10]. In addition, the female mutant mice with the absence of recognizable oocytes and follicles in the ovary resemble the clinical description of the sisters who were homozygous for the mutation [206]. A recent case report provided further support for the involvement of this gene in POI: a homozygous gross deletion affecting 4000 bp of *SYCE1* in two POI sisters have been identified in a highly consanguineous Chinese family [208].

***MSH4***: was found to be mutated in two Colombian sisters presenting with secondary amenorrhea [209]. Segregation analysis of the *MSH4* splicing variant in the family was consistent with a recessive mode of inheritance [209]. Its KO in mice leads to both female and male infertility secondary to defective chromosome synapsis during meiosis [210,211].

Interestingly, both NOA patients described by Krausz and colleagues [10] with variants in *SYCE1* and *MSH4* had at least one infertile sister, further supporting a common genetic origin for NOA and POI.

***C14orf39***: a homozygous frameshift mutation has been recently identified in a POI-affected patient from a consanguineous Pakistani family, in which two male siblings carrying the same variant presented with meiotic arrest [85]. This mutation is located in the N terminus of the protein, which contains two SYCE1 binding regions [85]. The mutant protein can still interact with SYCE1, but its ability to form aggregates with SYCE1 is diminished [85]. Importantly, *Six6os1* mutant female mice mimicked the POI phenotype of the affected sister, further confirming the pathogenic role of *C14orf39* both in male and female infertility [85,139].

***PSMC3IP*** and ***DMC1***: homozygous variants were reported in consanguineous families, in which the affected females presented with POI while the male proband had NOA [86,124].

***MEIOB***: a homozygous truncating mutation has been recently reported as the cause of POI in two sisters of a consanguineous family where the parents are double first cousins [134]. This *MEIOB* variant is expected to provoke meiotic defects and a depleted follicular stock, consistent with the phenotype of the *Meiob −/−* mouse that displays infertility in both sexes due to meiotic arrest [132,133].

***NR5A1***: in rare cases, sequence variants of the gene may result in POI [174,212], or in various disorders of gonadal development (DGD) or adrenal insufficiency. Notably, no genotype-phenotype correlation was observed with *NR5A1* variations. For instance, p.Gly146Ala, the most frequently described NR5A1 sequence variant, was detected in three 46, XY-DGD cases [213,214,215], in four POI [174,216,217], and in two infertile men [112,114]. Safari and colleagues [116] reported a case of two Iranian siblings affected by azoospermia and POI, due to the same heterozygous *NR5A1* mutation segregating in the family. Very recently, *NR5A1* variants have been reported in two families including individuals with 46, XY DGD and POI [218], further complicating the clinical significance of pathogenic *NR5A1* variants in a context of highly variable expressivity.

***SETX***: as described in males, homozygous mutations of the gene lead to a syndromic phenotype, including progressive ataxia and ovarian failure [219].

***WT1***: heterozygous missense and splicing variants have been associated with Frasier syndrome, a rare disease characterized by male pseudo-hermaphroditism and progressive glomerulopathy [220,221]. Mutated patients presented normal female external genitalia, streak gonads, XY karyotype and frequently developed gonadoblastoma. Glomerular symptoms arise during childhood and consist of proteinuria and nephrotic syndrome, progressing to end-stage renal failure in adolescence or early adulthood [220,221]. Apart from the syndromic manifestation, this gene has been reported both in male and in female as isolated cause of NOA and POI [121,122,222,223].

Interestingly, knockout of *Adad2*, *Terb1* and *Rad21l1* in mice leads to infertility in both sexes [138,210,224], suggesting a potential common role of these genes in NOA and POI phenotypes.

All these findings imply that a special attention has to be paid to female relatives of male patients with primary testicular failure, as approximately 37% (14/38) of NOA candidate genes are also implicated in either POI, female genital anomalies or complex phenotypes. In contrast to gonadal ambiguities and primary amenorrhea usually documented at birth and during puberty, respectively, POI is usually not recognizable until amenorrhea occurs. Thus, genetic counseling for NOA is of great relevance not only to the male family members but also to the female ones, in whom oocyte vitrification would allow fertility preservation before ovarian failure occurs.

## 5. Conclusions

For the last 40 years the diagnostic armamentarium for the detection of genetic factors involved in azoospermia has been restricted to a few routine tests such as Karyotype analysis, Y-chromosome microdeletions and the search for a few monogenic causes in selected cases of pre- and post-testicular azoospermia. Thanks to the widespread diffusion of WES, an increasing number of novel candidate genes of azoospermia have been identified, especially in CHH. In this disease, the yield of genetic testing is already over 50% (Figure 1). Large-scale exome sequencing in the frame of international networks, such as COST Action (BM1105) (https://www.chuv.ch/en/hhn/hhn-home/research/our-basic-scientists) and European reference network on rare endocrine conditions (Endo-ERN) (https://endo-ern.eu/), will further improve the molecular diagnosis of CHH. The clinical impact of discovering novel disease-causing genes in this condition is especially relevant for genetic counselling since the majority of these patients can generate their own biological child with the risk of transmission of the identified mutation(s).

Regarding the primary testicular failure, the missing genetic diagnosis is still high, accounting for about 70% of cases after the exclusion of all known acquired and genetic causes (Figure 1). Given the complexity of the spermatogenesis and the highly heterogeneous testicular phenotypes, only large exome and genome studies involving thousands of well-characterized patients have the potential to unravel recurrent genetic causes of NOA. Moreover, for this type of azoospermia, a major breakthrough is expected from ongoing consortia-based efforts. In fact, a growing number of novel candidate genes of MA were found and validated, thanks to the data sharing between different laboratories belonging to the International Male Infertility Genomics Consortium (IMIGC) (http://www.imigc.org) and to the Genetics of Male Infertility Initiative (GEMINI) consortium (http://www.gemini.conradlab.org) (see [225]). Similar to other medical fields, the major challenge in the monogenic diagnosis of NOA is represented by the attribution of a pathogenic role to the identified variants, especially if they are classified as VUSs. A possible solution of the issue seems to lie in the high-resolution phenotyping of candidate male infertility mouse mutants [226], thanks to the CRISPR-Cas9 technologies. This approach will indeed allow to overcome the difficulty in interpreting missense variants, demonstrating a cause-effect relationship between a given genotype and NOA phenotype. Very recently, genome sequencing in combination with single-cell RNA sequencing (scRNAseq) allowed to connect potential pathogenic mutations directly to the testicular cell type where the effect is likely to be exerted [81]. This technology could clarify the effect of potential disease-causing variants on the complex cellular structure of spermatogenesis.

Besides the Mendelian inheritance, other mode of transmission may underlie the NOA phenotype. As for CHH, which can also be explained by a digenic/oligogenic inheritance, the combined effect of two or more rare mutations in different candidate genes of NOA should be taken into consideration. Kasak and Laan showed a highly significant enrichment of active connections and complementary functions among loci implicated in NOA [11]. In this context, a possible scenario of digenicity/oligogenicity underlying the etiology of this complex and heterogeneous condition could be considered in the near future.

In this review we provided a brief description of those potential candidate genes which may be part of a gene panel-based diagnostic testing in the future. We classified these genes according to the associated testicular histology underlying the NOA phenotype. For some gene defects, the testis phenotype consistently shows pure SCOS/MA phenotypes, providing a pre-TESE prognostic value for the identified NOA-causing gene. Currently, the sole prognostic pre-TESE genetic test is based on the AZF deletion screening but, if these monogenic causes will be validated in large cohorts, the gene panel will complement AZF screening also as prognostic test for testicular sperm retrieval. The emerging data on shared genetic factors between NOA and POI will have a clinical impact both on family history taking and genetic counselling.

In the past three years we witnessed to a continuous increase of novel genetic factors causing NOA [11,225,227,228]. Although we expect to uncover many more candidate genes, we anticipate that besides mutations in protein coding genes, other genetic and epigenetic alterations may contribute to the NOA phenotype. Concerning the former, it is possible that some unanalyzed genetic alterations, such as synonymous single nucleotide variants and variants located in the regulatory regions (UTR), could be responsible for the loss of function of a NOA-associated gene. Concerning the epigenetic aspects, small and long non-coding RNAs are reported to have a regulatory role in spermatogenesis, potentially resulting in NOA [229,230]. In addition, environmental exposures and lifestyle factors could have an influence on the expression of genes involved in spermatogenesis [231]. Recently, significant changes in DNA methylation of spermatogenic cells have been observed in NOA patients, although further studies are needed to determine the impact of the epigenetic regulations on development of male infertility [232]. From a diagnostic point of view, genetic factors remain clinically the most relevant and we expect that a male infertility diagnostic gene panel will be available in the near future.

## Figures and Tables

**Table 1:** List of candidate genes involved in monogenic causes of Human NOA, divided according to the associated-testis histology: (**a**) SCOS phenotype, (**b**) maturation arrest phenotype, (**c**) different types of testicular phenotype (SCOS/MA/hypospermatogenesis) and (**d**) undefined testicular phenotype. Detailed genomic and clinical information, the female and male mouse reproductive phenotypes and references are reported.

**(a)**											
**Gene ^**	**OMIM**	**Locus °**	**Function ^+^**	**Inheritance**	**Other Phenotypes**	**POI**	**Mouse Reproductive Phenotypes ^#^**	**Segregation in Family**	**More than One Unrelated Carrier**	**Independent Cohorts**	**Refs.**
*FANCA*	607139	16q24.3	Interstrand crosslink repair	AR	Fanconi Anemia	Yes	Abnormal male meiosis, decreased germ cell number, decreased mature ovarian follicle number, absent ovarian follicles	Yes	Yes	No	[82]
*PLK4*	605031	4q28.1	Centriole duplication during the cell cycle	AD	Microcephaly and chorioretinopathy	No	Decreased male germ cell number	No	No	No	[83]
*WNK3*	300358	Xp11.22	Regulation of electrolyte homeostasis, cell signaling, survival and proliferation	XLR	n.r.	No	Normal *	Yes	No	No	[84]
**(b)**											
**Gene ^**	**OMIM**	**Locus °**	**Function ^+^**	**Inheritance**	**Other Phenotypes**	**POI**	**Mouse Reproductive Phenotypes ^#^**	**Segregation in Family**	**More than One Unrelated Carrier**	**Independent Cohorts**	**Refs.**
*ADAD2*	n.a.	16q24.1	dsRNA-binding protein, RNA editing	AR	n.r.	No	Male and female infertility	Yes	Yes	Yes	[10]
*C14orf39*	617307	14q23.1	Chromosome synapsis during meiotic recombination	AR	n.r	Yes	Arrest of male meiosis, abnormal chiasmata formation, abnormal chromosomal synapsis, abnormal X-Y chromosome synapsis during male meiosis, absent oocytes	Yes **	Yes	No	[85]
*DMC1*	602721	22q13.1	Meiotic recombination, DNA DSB repair	AR	n.r.	Yes	Arrest of male meiosis decreased oocyte number, absent oocytes, absent ovarian follicles, abnormal female meiosis	Yes **	No	No	[86]
*KASH5*	618125	19q13.33	Meiotic telomere attachment to nuclear envelope in the prophase of meiosis, homolog pairing during meiotic prophase	AR	n.r.	No	Arrest of male meiosis, female infertility	Yes	No	No	[84]
*MAJIN*	617130	11q13.1	Meiotic telomere attachment to the nucleus inner membrane during homologous pairing and synapsis	AR	n.r.	No	Meiotic arrest, male and female infertility	No	No	No	[87]
*MEI1*	608797	22q13.2	Meiotic chromosome synapsis, DBS formation	AR	Hydatidiform mole	Yes	Arrest of male meiosis, female infertility	Yes	Yes	Yes	[10,88,89]
*MEIOB*	617670	16p13.3	DNA DSB repair, crossover formation and promotion to complete synapsis	AR	n.r.	Yes	Arrest of spermatogenesis, decreased oocyte number, absent oocytes	Yes	Yes	Yes	[10,90,91]
*MSH4*	602105	1p31.1	Homologous chromosomes recombination and segregation at meiosis I	AR	n.r.	Yes	Azoospermia, abnormal male and female meiosis	No	Yes	Yes	[10]
*RAD21L1*	n.a.	20p13	Meiosis-specific component of some cohesin complex	AR	n.r.	No	Arrest of male meiosis, absent oocytes, decreased mature ovarian follicle number, absent primordial ovarian follicles	Yes	No	No	[10]
*RNF212*	612041	4p16.3	Regulator of crossing-over during meiosis	AR	n.r.	No	Arrest of male meiosis, female infertility	Yes	No	No	[92]
*SETX*	608465	9q34.13	DNA and RNA processing	AR	Amyotrophic lateral sclerosis; ataxia with oculomotor apraxia type 2	Yes	Arrest of male meiosis, globozoospermia, reduced female fertility	No	Yes	Yes	[93,94]
*SHOC1*	618038	9q31.3	Binds to single-stranded DNA and DNA branched structures; formation of crossover recombination intermediates	AR	n.r.	No	Arrest of male meiosis	Yes	Yes	Yes	[10,95]
*SPINK2*	605753	4q12	Inhibitor of acrosin	AR	n.r.	No	Kinked sperm flagellum, oligozoospermia, teratozoospermia, abnormal male germ cell apoptosis	Yes	No	No	[1,96]
*SPO11*	605114	20q13.31	Initiation of DSBs	AR	n.r.	No	Arrest of male meiosis, decreased oocyte number, oocyte degeneration, abnormal female meiosis	Yes	No	No	[84]
*STAG3*	608489	7q22.1	Cohesion of sister chromatids, DNA DSB repair	AR	n.r.	Yes	Azoospermia, absent oocytes	Yes **	Yes	Yes	[10,92,97,98]
*STX2*	132350	12q24.33	Sulfoglycolipid transporter	AR	n.r.	No	Arrest of male meiosis	No	No	No	[99]
*SYCE1*	611486	10q26.3	Chromosome synapsis in meiosis	AR	n.r.	Yes	Arrest of male meiosis, decreased mature ovarian follicle number	Yes	Yes	Yes	[10,100,101]
*TDRD7*	611258	9q22.33	RNA processing	AR	Congenital cataract	No	Arrest of spermatogenesis, abnormal male germ cell apoptosis	Yes	Yes	No	[2,102]
*TERB1*	617332	16q22.1	Meiotic telomere attachment to the nucleus inner membrane during homologous pairing and synapsis	AR	n.r.	No	Arrest of male meiosis, absent oocytes, absent ovarian follicles, abnormal female meiosis I arrest	Yes	Yes	Yes	[10,87]
*TERB2*	617131	15q21.1	Meiotic telomere attachment to the nucleus inner membrane during homologous pairing and synapsis	AR	n.r.	No	Arrest of male meiosis, absent ovarian follicles, abnormal female meiosis	Yes	No	No	[87]
*TEX11*	300311	Xq13.1	Chromosome synapsis and formation of crossovers	XLR	n.r.	No	Arrest of male meiosis, meiotic non-disjunction during M1 phase	Yes	Yes	Yes	[10,103,104,105]
*XRCC2*	600375	7q36.1	Interstrand crosslink repair, DNA DSB repair	AR	Fanconi Anemia	Yes	Meiotic arrest, POI	Yes **	No	No	[106]
*ZMYND15*	614312	17p13.2	Transcriptional repressor	AR	n.r.	No	Azoospermia	Yes	No	No	[107]
**(c)**											
**Gene ^**	**OMIM**	**Locus °**	**Function ^+^**	**Inheritance**	**Other Phenotypes**	**POI**	**Mouse Reproductive Phenotypes ^#^**	**Segregation in Family**	**More than One Unrelated Carrier**	**Independent Cohorts**	**Refs.**
*DMRT1*	602424	9p24.3	Transcription factor involved in male sex determination and differentiation	AD	Ambiguous genitalia and sex reversal	No	Abnormal male meiosis, male infertility	Yes	Yes	Yes	[10,108]
*FANCM*	609644	14q21.2	DNA DSB repair, interstrand cross-link removal	AR	n.r.	Yes	Azoospermia, decreased mature ovarian follicle number	Yes	Yes	Yes	[109,110]
*M1AP*	619098	2p13.1	Meiosis I progression	AR	n.r.	No	From arrest of male meiosis to severe oligozoospermia/globozoospermia	Yes	Yes	Yes	[111]
*NANOS2*	608228	19q13.32	Spermatogonial stem cell maintenance	AR	n.r.	No	Azoospermia, abnormal female meiosis	Yes	Yes	No	[84]
*NR5A1*	184757	9q33.3	transcriptional activator for sex determination	AD	46, XY and 46, XX sex reversal; adrenocortical insufficiency	Yes	From oligozoospermia to arrest of spermatogenesis, decreased mature ovarian follicle number, absent mature ovarian follicles	Yes **	Yes	Yes	[112,113,114,115,116]
*TAF4B*	601689	18q11.2	Transcriptional coactivator	AR	n.r.	No	Oligozoospermia, decreased male germ cell number, asthenozoospermia, absent mature ovarian follicles, impaired ovarian folliculogenesis	Yes	No	No	[107]
*TDRD9*	617963	14q32.33	Repression of transposable elements during meiosis	AR	n.r.	No	Arrest of male meiosis	Yes	No	No	[117]
*TEX14*	605792	17q22	Formation of meiotic intercellular bridges	AR	n.r.	No	Arrest of male meiosis	Yes	Yes	Yes	[10,84,90]
*TEX15*	605795	8p12	Chromosome, synapsis, DNA DSB repair	AR	n.r.	No	Arrest of male meiosis	Yes	Yes	Yes	[118,119]
*WT1*	607102	11p13	Transcription factor	AD	Wilms tumor type 1; Nephrotic sdr type 4; Denys-Drash sdr; Frasier sdr; Meacham sdr; Mesothelioma	Yes	Azoospermia	No	Yes	Yes	[120,121,122]
**(d)**											
**Gene ^**	**OMIM**	**Locus °**	**Function ^+^**	**Inheritance**	**Other Phenotypes**	**POI**	**Mouse Reproductive Phenotypes ^#^**	**Segregation in Family**	**More than One Unrelated Carrier**	**Independent Cohorts**	**Refs.**
*MCM8*	608187	20p12.3	DNA DSB repair, interstrand crosslink removal	AR	n.r.	Yes	Arrest of male meiosis, decreased oocyte number, decreased mature ovarian follicle number, increased ovary tumor incidence, increased ovary adenoma incidence	Yes **	No	No	[123]
*PSMC3IP*	608665	17q21.2	Stimulating DMC1-mediated strand exchange required for pairing homologous chromosomes during meiosis.	AR	Ovarian dysgenesis	Yes	Arrest of male meiosis, absent ovarian follicles, abnormal ovary development	Yes **	No	No	[124]

^ based on HGNC; **°** according to Human GRCh38/hg38; ^+^ based on GeneCards database; ^#^ male and female mouse reproductive phenotypes based on MGI database; * *Wnk3* −/− females from *Wnk3* Y/− fathers were reported [125], although the mouse male reproductive phenotype was not investigated (subtle spermatogenic impairment cannot be excluded); ** cosegregating in family with NOA and POI phenotypes. n.r. = not reported; n.a. = not available; POI = Primary Ovarian Insufficiency; DBS = double-strand break; sdr = syndrome; XLR = X-linked recessive.

## Data Availability

Please refer to suggested Data Availability Statements in section “MDPI Research Data Policies” at https://www.mdpi.com/ethics.
